# Treatment of Acute Pulmonic Inflammations in Young Children

**Published:** 1888-02

**Authors:** Q. C. Smith

**Affiliations:** Austin, Texas


### THE TREATMENT OF ACUTE PULMONIC INFLAMMATIONS OF YOUNG CHILDREN.

*By Q. C. Smith, M. D., Austin, Texas.*

(Read before the Travis County Medical Society, January 5, 1888. By invitation of the Society.)

For Daniel's Texas Medical Journal.

OWING to the anatomical peculiarities of the respiratory organs of young children, it has been found necessary to somewhat modify the treatment, such as is usually adapted to adults in the treatment of acute inflammatory diseases of the respiratory organs of children under the age of one or two years.

To be brief, practical and to the point, we will refer to the treatment of this class of diseases collectively, as in many cases the treatment of each form of this class of diseases, is more or less similar, even when a well defined differential diagnosis can be made—which is not always easy or even possible to establish.

We will consider the treatment of bronchitis. catarrhal pneumonia, and pleurisy. Capillary bronchitis being usually a result of the tubular form, and catarrhal pneumonia usually a result of capilliary bronchitis.

Pure croupous pneumonia—the more common form in adults—is rare in young children.

Acute pleurisy, which is not so frequent or fatal as bronchitis or catarrhal pneumonia or their combinations in infants, yet is more frequent than is usually recognized, and should be carefully sought for in all cases of acute pulmonic inflammation.

Having briefly ennumerated the three leading acute idiopathic pulmonic inflamatory diseases that usually affect young children, we will now proceed to mention some of the recognized means and measures we more or less frequently use for their relief or cure.

There are two cardinal points which have an important bearing in reference to the treatment which we desire to call forcibly to your rememberance in regard to this class of diseases:

1. They are, in our opinion, all *essential* fevers, *i. e.* the febrile movement accompanying their course is not a mere reactionary pyrexia, resulting from a traumatic lesion of the parts involved, but an essential fever in the same sense as we so speak of typhoid and typhus fevers.

2. It therefore follows, as a well known pathological concatenation, that should we be able to so modify the course of the disease and mitigate its severity and obviate the tendency to death by judiciously conserving the vital powers, we may often conduct the patient from a very grave condition to a full restoration of health, with the use of only a small quantity of drug remedies.

A tedious repetition of what standard works and periodical literature say of the treatment of this class of diseases, would be a waste of time, unprofitable and presumptious to say the least of it.

Furthermore, to tell at wearisome length just what or how I would probably do in the treatment of all the various and varied real or hypothetical phases, stages and conditions that might arise in what we would denominate bronchitis, pneumonia or pleurisy, would be a useless waste of time and effort; therefore, the means, measures and remedies we may mention, must be understood to be of more or less general application.

One point we should never fail to attend to during our first visit to a case of this class of diseases is to see that the wise admonition of the immortal Boerhaave, as laid down in his great unwritten volume, is sedulously carried out—that the patient's head be kept cool, feet warm and bowels open. For with all our boasted skill in modern physical diagnosis we are, therapeutically, not very far ahead of the great English Hippocrates, who said more than two hundred years ago that it was of little practical consequence whether we could accurately differentiate the acute pulmonic inflammations or not, as their treatment was usually quite similar.

In most all cases of acute pulmonic inflammation of babes, unless there be marked manifestations of cerebral disturbance, we hasten to bring the patient under the influence of quinine which we usually administer in the form of bimuriate, either per rectum or endermatically. When we administer the bimuriate of quinine to babes, per rectum or endermatically, we have the salt mixed thoroughly with lanolin; and if per rectum, put in hollow cocoa butter capsules; by the skin, we have the medicament rubbed over the chest until it is all absorbed. Dose, by these methods, one to three grains, to be repeated every three to twelve hours.

If called to treat a bade suffering from a severe acute attack of bronchitis or pneumonia within the first twenty-four hours, and the patient be not extremely debilitated, we usually administer an emetic of ipecac, and if there be pleurisy add a portion of a drop of Flemming's tinct. aconite to each dose of the emetic until emesis is produced. The emetic should be well diluted with warm water, made sweet with liquorice or sugar, and given in broken doses, at intervals of fifteen to thirty minutes until effective. We rarely repeat the emetic course in any case.

Should the patient not be greatly prostrated we follow the emetic course with one-twelfth to one-sixth of a grain of calomel, well mixed with milk sugar, one or two doses only. If the bowels are not moving freely, we follow this with some pleasant purgative in small doses, well diluted, such as cascara cordial or sweet senna tea, until the bowels move freely, then only enough afterwards to keep the bowels in a soluable condition, moving one to three times every twenty-four hours. To relieve cough we usually give syrups of senega, lactucarium, ipecac and wild cherry, dilute and in small doses.

Early in these cases, especially if there is a marked degree of asthenia, a very warm bath, with plenty of rubbing, ten minutes duration, is often a great benefit. If there be much pain, mild counter-irritation, produced by rubbing the chest with a liniment composed of hydrate chloral, camphor, fl: ex. aconite rad, well mixed with lanolin, to be applied one to three times a day. Later on in the case, or if asthenic, early, we give carbonate and muriate of ammonia in small doses, pleasantly diluted. As specific alteratives the following are favorites with us: Asclepias tuberosa, bryonia, senega, ergot and ipecac; the ergot we deem most useful in the early stages—all these to be given in very small doses, well diluted with plain or sweet water. If the heart-beats are very rapid and weak, with dispnœa, or cyanosis be manifest, we give tinct. strophanthus, sometime with tinct. digitalis, in one-twentieth to one-fiftieth of a drop, well diluted, every two to four hours, until these symptoms are relieved.

A warm moist woolen-flannel girdle to cover the whole chest, especially in the early stages, often gives great comfort, provided, the nurse be skilful and attentive. We use as little opium as possible—often none—in the treatment of this class of diseases, and if any, prefer svapnia and codeia.

The pains, even of acute pleurisy, can almost always be relieved by very small doses of tinct. aconite, with anodyne liniments and very warm applications to the surface. If the larynx, or pharynx be involved, we give nitrate of sanguinariana, dilute, in very small doses.

Our judgment directs, that, with rare exceptions, all medicines for babes, should be given well diluted in very small doses, and made pleasant to take.

As these cases progress we endeavor to meet each symptom or condition that may arise, as best we may, constantly studying the comfort of the patient, with such remedies, means and measures, as are, or should be, familiar to all intelligent physicians, who have given special attention to the treatment of sick babies. The general hygiene, and judicious feeding of these tender patients, is of the first importance in all cases and is paramount to the administration of drugs. Sick babies—and well ones too—are usually fed too often; every three hours is often enough, and even longer intervals, in many cases, will produce better results.

